# Establishment and validation of a novel peroxisome-related gene prognostic risk model in kidney clear cell carcinoma

**DOI:** 10.1186/s12894-024-01404-z

**Published:** 2024-01-31

**Authors:** Jing Zhang, Qian Zhao, Hongwei Huang, Xuhong Lin

**Affiliations:** 1 School of Stomatology, Henan University, Jinming Road, Kaifeng, Henan 475000 China; 2 Department of Pediatric General Surgery, The Third Affiliated Hospital of Zhengzhou University, No. 7 Kangfu Qian Street, Zhengzhou, Henan 450052 China; 3 Department of Clinical Laboratory, Huaihe Hospital of Henan University, No.115 Ximen Street, Kaifeng, Henan 475000 China

**Keywords:** Kidney clear cell carcinoma, Peroxisome-related genes, Prognosis, Immune infiltration

## Abstract

**Background:**

Kidney clear cell carcinoma (KIRC) is the most common subtype of renal cell carcinoma. Peroxisomes play a role in the regulation of tumorigenesis and cancer progression, yet the prognostic significance of peroxisome-related genes (PRGs) remains rarely studied. The study aimed to establish a novel prognostic risk model and identify potential biomarkers in KIRC.

**Methods:**

The significant prognostic PRGs were screened through differential and Cox regression analyses, and LASSO Cox regression analysis was performed to establish a prognostic risk model in the training cohort, which was validated internally in the testing and entire cohorts, and further assessed in the GSE22541 cohort. Gene Ontology (GO) enrichment and Kyoto Encyclopedia of Genes and Genomes (KEGG) pathway analyses were performed to explore the function and pathway differences between the high-risk and low-risk groups. The relationship between risk score and immune cell infiltration levels was evaluated in the CIBERSORT, ESTIMATE and TIMER databases. Finally, potential biomarkers were identified and validated from model genes, using immunohistochemistry.

**Results:**

Fourteen significant prognostic PRGs were identified using multiple analyses, and 9 genes (ABCD1, ACAD11, ACAT1, AGXT, DAO, EPHX2, FNDC5, HAO1, and HNGCLL1) were obtained to establish a prognostic model via LASSO Cox regression analysis. Combining the risk score with clinical factors to construct a nomogram, which provided support for personalized treatment protocols for KIRC patients. GO and KEGG analyses highlighted associations with substance metabolism, transport, and the PPAR signaling pathways. Tumor immune infiltration indicated immune suppression in the high-risk group, accompanied by higher tumor purity and the expression of 9 model genes was positively correlated with the level of immune cell infiltration. ACAT1 has superior prognostic capabilities in predicting the outcomes of KIRC patients.

**Conclusions:**

The peroxisome-related prognostic risk model could better predict prognosis in KIRC patients.

**Supplementary Information:**

The online version contains supplementary material available at 10.1186/s12894-024-01404-z.

## Background

Renal cell carcinoma (RCC) is one of the most common malignancies in the world [1], comprising three primary types: kidney renal clear cell carcinoma (KIRC), which accounts for approximately 75% of all renal cancers; papillary renal cell carcinoma (pRCC); and chromophobe cell carcinoma (ChRCC) [2, 3]. What’s more, in contrast to pRCC and ChRCC patients, KIRC patients often face a worse prognosis and heightened propensity for metastasis. The early symptoms are subtle, while the pathogenesis in advanced stages is intricately multifaceted, contributing to metastases occurring in 20-30% of patients upon tumor detection [4]. The initial treatment for KIRC typically involves partial or radical nephrectomy, yet about 30% of patients experience postoperative recurrence [5], and advanced therapeutic ways, including molecular targeted therapy and immunotherapy, have been employed, but their outcomes remain less than optimal. Currently, the etiology of KIRC remains unidentified, and the absence of an effective prognostic prediction model further compounds the clinical challenge. Consequently, the identification of biomarkers and the construction of a reliable model hold crucial clinical significance for enhancing the prognostic evaluation of KIRC.

The peroxiredoxin family, widely distributed across prokaryotes and eukaryotes, stands out as crucial antioxidants with peroxide-scavenging activity, and peroxisomes are predominantly found in hepatocytes and renal proximal tubular epithelial cells in mammalian tissues. Peroxisomes have been shown to be effective in destroying hydrogen peroxide produced by metabolism [6]. What’s more, recent research has highlighted the pivotal role of peroxisomes in the tumorigenesis and progression of KIRC [7]. Furthermore, peroxisomes actively participate in lipid metabolism and the peroxisomal-oxidation system involves the metabolism of long-chain acyl-coenzyme A (acyl-CoA), generating H_2_O_2_ as a byproduct [8]. Numerous studies have consistently reported the upregulation of various peroxisomal proteins in tumors, showcasing associations with tumor stage, infiltration, recurrence, and prognosis. Notably, peroxisome-related genes have been identified as regulators of tumor progression, and their significance in the intricate landscape of cancer biology were underscored [9].

**Fig. 1:**
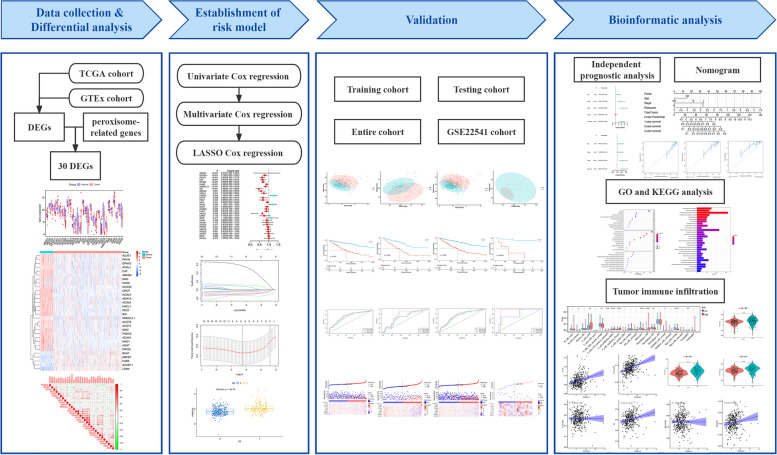
Workflow diagram

Given the phenomenon, our study focused on establishing a peroxisome-related gene prognostic risk model, which was validated through internal and external cohorts. The independent prognostic ability was evaluated by univariate and multivariate Cox regression analyses. Additionally, we delved into the functional, pathway and immune landscape differences between high-risk and low-risk groups using various analyses, providing a comprehensive understanding of the molecular landscape associated with peroxisome-related genes in KIRC. Finally the potential prognostic biomarkers were identified from the model genes and validated using immunohistochemistry (IHC), and the workflow diagram was shown in Fig. 1.

## Materials and methods

### Dataset collection

The RNA sequencing and corresponding clinical data for 536 KIRC and 72 normal samples were downloaded from UCSC Xena (http://xena.ucsc.edu/). We also downloaded the GSE22541 cohort, including 24 samples, for external validation from the GEO (https://www.ncbi.nlm.nih.gov/) database.

Criteria for data: (1) only patients with primary KIRC were included; (2) only samples with complete RNA sequencing data were included. Exclusion Criteria for data: (1) patients with recurrent KIRC; (2) samples with survival time of 0. The TCGA-KIRC cohort included 522 KIRC samples and 24 normal samples were selected in the GSE22541 cohort.

The 522 KIRC samples in the TCGA-KIRC cohort were randomly divided into the training cohort (70%, *n* = 365) and testing cohort (30%, *n* = 157) using 10-fold cross-validation. Table S1 presented KIRC patients’ clinical data of above cohorts. The PRGs were extracted from previous literature [10], Kyoto Encyclopedia of Genes and Genomes (KEGG, https://www.genome.jp/kegg/) and Gene Set Enrichment Analysis (GSEA, https://www.gsea-msigdb.org/gsea/index.jsp) databases, of which 113 genes were shown in Table S2.

### Differential analysis

The differential analysis between 365 KIRC and 72 normal samples in the training cohort was performed to screen differentially expressed peroxisome-related genes (DE-PRGs), utilizing the “limma” package [11], and the selection criteria were |log_2_Foldchange|>1 and *p* < 0.05.

### Cluster analysis

To explore the relationship between DE-PRGs and KIRC subtypes, the “ConsensusClusterPlus” package [12] was employed to perform cluster analysis based on Sangerbox (http://sangerbox.com/). Agglomerative PAM clustering with 1-Pearson correlation distances was applied, and 80% of the samples were resampled for ten repetitions. The optimal number of clusters was determined using the empirical cumulative distribution function plot.

### Establishment of a peroxide-related gene risk model in the training cohort

The important prognostic significance of DE-PRGs was evaluated by using univariate Cox regression analysis in the training cohort, and subsequently combined with important clinical factors, including age, grade and stage, the multivariate Cox regression analysis was performed to further assess the prognostic value. Ultimately, the candidate DE-FRGs were narrowed down using LASSO Cox regression analysis to establish a peroxide-related gene risk model. The risk formula was presented, and the risk score =${\sum }_{i=1}^{n}coef*geneexpression$. According to the optimal cutoff value, the KIRC patients in the training cohort were divided into high-risk and low-risk groups, and the difference of survival status between two subgroups was analyzed and compared through Kaplan‒Meier method and log-rank test. The sensitivity and specificity of the gene risk model were evaluated using time-dependent receiver operating characteristic (ROC) curves, and the principal component analysis (PCA) was performed to detect differences of risk model genes expression patterns of two subgroups.

### Validation and evaluation of a peroxide-related gene risk model in the testing, entire and GSE22541 cohorts

In order to validate the general applicability of the prognostic efficacy of the gene risk model, the KIRC patients were classified into two categories in the testing, entire and GSE22541 cohorts. Furthermore, PCA, Kaplan-Meier and ROC curves were performed to validate the accuracy of the gene risk model.

### Independent prognostic analysis

To evaluate the clinical applicability of the risk model, we conducted univariate and multivariate Cox regression analyses which aimed to ascertain whether the risk score derived from the risk model in the training cohort could be considered as an independent prognostic factor.

### Establishment and validation of the nomogram

Utilizing the ''rms'' package, we integrated survival time, survival status, and significant clinical factors identified through Cox analyses, along with the risk score derived from the risk model, to construct a comprehensive nomogram. This nomogram was designed to predict the overall survival (OS) of KIRC patients at 1, 2, and 3 years. To assess the predictive accuracy of the nomogram, we employed calibration curves and the C-index as evaluation metrics.

### Functional enrichment analysis

To further analyze the difference of biological functions and pathways of two subgroups, which were stratified in the entire cohort utilizing the optimal cutoff value derived from the risk score, we conducted Gene Ontology (GO) enrichment analysis and KEGG pathway analysis based on the DEGs, employing stringent criteria (|log_2_fold change|>1, *p* < 0.05), identified between the high-risk and low-risk groups.

### Tumor immune infiltration

To investigate the immune status between different risk groups, we first used CIBERSORT (https://cibersort.stanford.edu/) database to calculate the samples of 22 kinds of immune cell infiltration scores. The ESTIMATE algorithm(https://bioinformatics.mdanderson.org/estimate/) was used to predict the immune score and stromal score for each sample. The abundance of 6 tumor immune cell infiltrations in KIRC was analyzed by TIMER (https://cistrome.shinyapps.io/timer/) database Spearman correlation analysis was used to evaluate the correlation between immune cells and risk scores, and to explore the correlation between the expression of 9 genes and the abundance of immune infiltrations by using its “Gene” module. The “SCNA” module was used to explore the correlation between somatic copy number alterations for 9 genes and the abundance of immune infiltration in the TIMER database.

### Identification of potential prognostic biomarkers

The OS and recurrence-free survival (RFS) Kaplan-Meier curves of 9 risk model genes were generated, which proved instrumental in evaluating the potential prognostic value of these risk model genes in predicting prognosis for KIRC patients, using the GEPIA2.0 (http://gepia2.cancer-pku.cn/#index) database. To gain a more comprehensive understanding of the expression of these genes in KIRC, we conducted assessments at both mRNA and protein levels using UALCAN (https://ualcan.path.uab.edu/index.html) and HPA (https://www.proteinatlas.org/) databases.

### Immunohistochemistry

Paraffin tissue samples of 2 KIRC and 2 normal achival specimens from the Pathology Department of Huaihe Hospital of Henan University in 2023 were collected. To guarantee that the thickness of each section was the same, and that other contributing elements were consistent, section work was carried out by the same pathological experimenter. Tissues were fixed in 4% paraformaldehyde at room temperature overnight. After gradient alcohol dehydration, parafffn embedded tissues were sliced into sections (5 μm thick), and were subjected to dewaxing (60˚C for 2 h), followed by soaking in dimethylbenzene twice for 15 min, hydration, antigen retrieval and washing with PBS. 3% H_2_O_2_ was used to block the endogenous peroxidase for 10 min at room temperature, and the slides were incubated with anti ACAT1 (1:50; cat. no. 16215-1-AP; Proteintech) antibody dissolved in blocking solution (QuickBlock™; Beyotime Institute of Biotechnology) at 4˚C overnight. After incubation, the slides were washed with PBS and incubated with HRP labelled polymer system (cat. no. E-IR-R215; Elabscience Biotechnology Co, Ltd) at 37˚C for 15 min, followed by an incubation with 3,3'- diaminobenzidine(DAB) detection reagent at room temperature for 5 min, and finally observed under light microscope with a magnification of x200. The semi quantitative expression of each protein was analyzed by Image Pro Plus software v.6.0 (Media Cybernetics, Inc.).

### Statistical analysis

All statistical analyses were conducted by R version 4.1.1, other unused ones had been specifically noted.

## Results

### Difference analysis between 365 KIRC and 72 normal samples

**Fig. 2:**
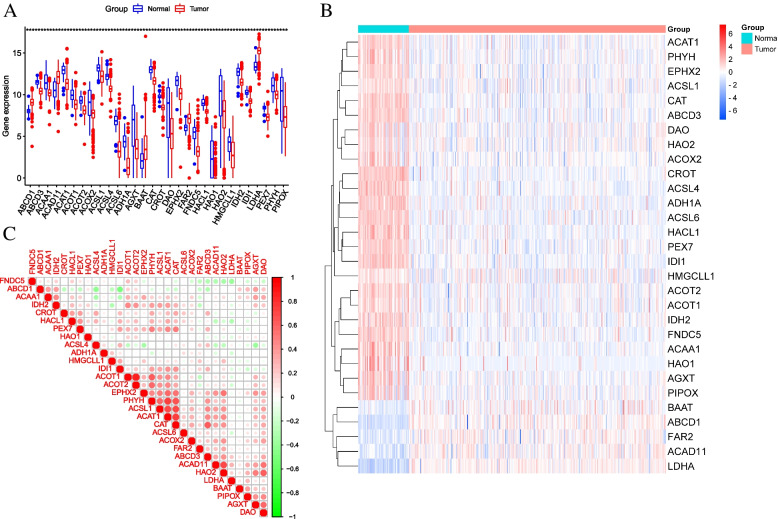
Identification of DE-PRGs between KIRC and normal tissues. **A** 30 DE-PRGs expression difference between KIRC and normal tissues (*p< 0.05; **p< 0.01; ***p< 0.001). **B** The heatmap of 30DE-FRGs expression level in KIRC and normal tissues (blue: low expression; red: high expression). **C** Interaction analysis among the 30 DE-PRGs (green: negative correlation; red: positive correlation)

Through differential analysis, we identified 30 DE-PRGs (Fig. 2A), of them the expression of 5 genes (ABCD1, FAR2, ACAD11, LDHA, BAAT) was upregulated, while the expression of 25 other genes (ABCD3, ACSL1, ACSL4, ACSL6, ACAT1, ACCAA1, ACOT1, ACOT2, ACOX2, ADH1A, AGXT, CAT, DAO, EPHX2, FNDC5, HACL1, HAO1, HAO2, CROT, IDH2, HMGCLL1, IDI1, PHYH, PIPOX, IDI1) was downregulated. The expression levels of these genes were shown in Fig. 2B. We also analyzed the correlation between 30 genes and found that most genes showed a positive correlation, with ACOT2 and ACOT1 being the most relevant (Fig. 2C).

### Classification of KIRCs based on 30 DE-PRGs

**Fig. 3:**
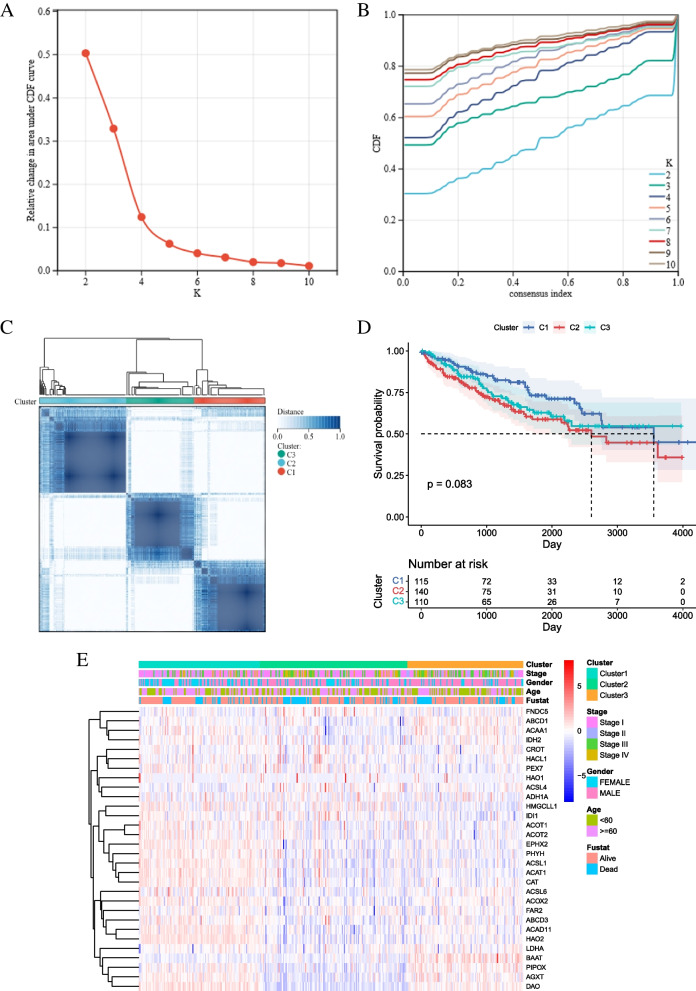
Clinical factors and survival status of KIRC among cluster1, cluster2 and cluster3 subtypes in the training cohort. **A** Relative change in the area under the CDF curve for k = 2–10. **B** Consensus clustering cumulative distribution function (CDF) for k = 2 to 10. **C** The training cohort was grouped into three clusters according to the consensus clustering matrix k = 3. **D** Survival curves for the three clusters. (E)Heatmap and distribution of the clinicopathologic characters of the three clusters classified by these peroxisome-related genes

To explore the relationship between 30 DE-PRGs expression and KIRC subtypes, the patients in the training cohort were grouped through cluster analysis. The area under the CDF curve gradually increased when the K value increased. On the premise of keeping the area under the curve as large as possible, according to the CDF Delta downward trend assessment, the delta decline was kept at the slowest pace, and the number of clusters was selected based on the combination of the above two factors. The optimal number of clusters was K = 3, and the number of suboptimal clusters was K = 2 (Fig. 3A-C). Through survival analysis, we found that the OS of the three groups was not significantly different (Fig. 3D, *p* > 0.05). The clinical factors of the tumor, including stage (Stage I-IV), age (< 60 or ≥ 60 years old), and fustat (alive or dead), were shown by a heatmap, and there was no statistically significant difference (Fig. 3E).

### Establishment and validation of the peroxisome-related risk model in the TCGA and GSE22541 cohorts

**Fig. 4:**
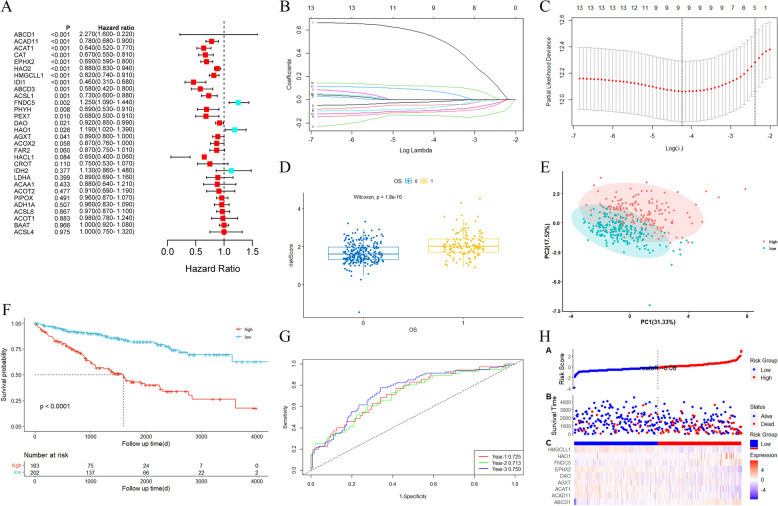
Establishment of a peroxisome-related gene risk model in the training cohort. **A** Univariate Cox regression analysis of OS for 30 DE-PRGs. **B** LASSO Cox regression analysis of the 14 DE-PRGs. **C** Cross-validation for tuning the parameter selection in the LASSO Cox regression analysis. **D** The boxplot showed the distribution of risk scores for survival and death patients. **E** PCA plot for KIRCs based on the risk score of the training cohort. **F** Survival curves for patients in the high-risk and low-risk groups of the training cohort. **G** ROC demonstrated the predictive efficiency of the risk score of the training cohort. **H** Distribution of patients based on the risk score of the training cohort. (up). The survival status for each patient of the training cohort. (low-risk population: on the left side of the dotted line; high-risk population: on the right side of the dotted line) (mid). Heat map of patient gene signature based on risk score of the training cohort. (down)

Sixteen prognostic genes (ABCD1, ACAD11, ACAT1, CAT, EPHX2, HAO2, HMGCLL1, IDI1, ABCD3, ACSL1, PHYH, PEX7, DAO, HAO1, FNDC5, AGXT, *p* < 0.05) were screened through univariate Cox regression analysis (Fig. 4A). Subsequently, by combining 16 genes with significantly prognostic clinical factors, including age, grade and stage, 14 significantly prognostic genes (ABCD1, ACAD11, ACAT1, CAT, EPHX2, HAO2, HMGCLL1, ABCD3, ACSL1, PHYH, DAO, HAO1, FNDC5, AGXT, *p* < 0.05) were identified by multivariate Cox regression analysis (Fig. S1). Finally, 9 genes were selected to establish a gene risk model via LASSO Cox regression, and the optimal λ value was 0.0173 (Fig. 4B-D). The risk score= (0.570 * ABCD1 exp.) + (− 0.046 * ACAD11 exp.) + (− 0.135 * ACAT1 exp.) + (− 0.073 * AGXT exp.) + (− 0.021 *DAO exp.) + (− 0.108 * EPHX2 exp.) + (0.118*FNDC5 exp.) + (0.083*HAO1exp.) + (-0.094 *HMGCLL1 exp.). According to the optimal cutoff value (cut off = 1.848551) of the risk score, the training cohort was divided into high-risk and low-risk groups, and PCA indicated that patients could be well divided into two subgroups (Fig. 4E). There was a statistically significant difference in overall survival time and rate between two subgroups, the high-risk group had more deaths and shorter survival time than the low-risk group (*p* < 0.01, Fig. 4F and H). The area under the ROC curves (AUC) of 1-, 2- and 3- year were 0.725, 0.713 and 0.750 in the training cohort, respectively (Fig. 4G).

**Fig. 5:**
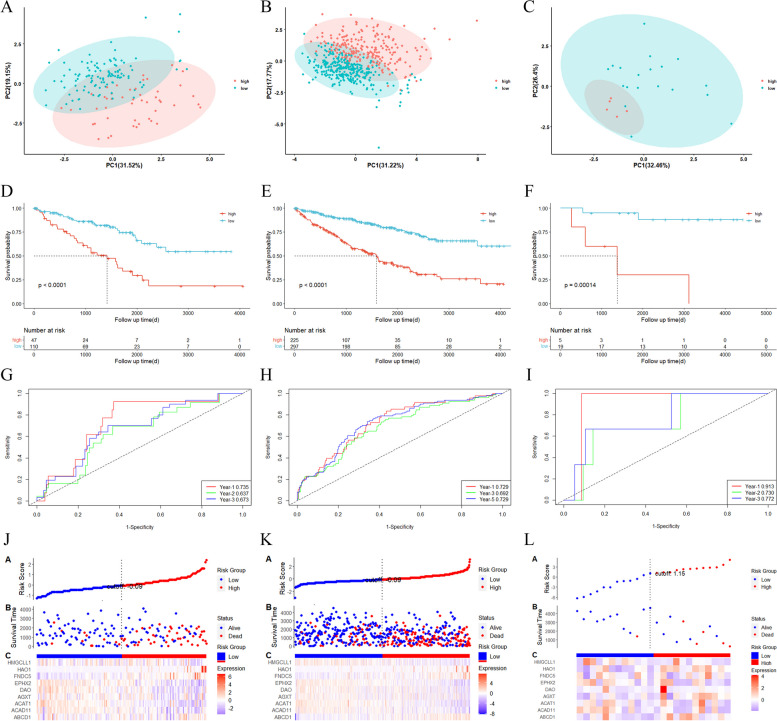
Internal and external validation of the peroxisome-related gene risk model. **A**-**C** PCA plots for KIRCs based on the risk score of the testing, entire and GSE22541 cohorts. **D**-**F** Kaplan–Meier curves for the OS of patients in the high-risk and low-risk groups of testing, entire and GSE22541 cohorts. **G**-**I** ROC indicated the predictive efficiency of the risk score of the testing, entire and GSE2254 cohorts. **J**-**L** Distribution of patients based on the risk score of the testing, entire and GSE2254 cohorts (up). The OS for each patient of the testing, entire and GSE22541cohorts. (low-risk population: on the left side of the dotted line; high-risk population: on the right side of the dotted line) (mid). Heat map of risk model based on risk score of the training cohort. (down)

Based on the optimal cutoff risk score, the KIRC patients were classified into two subgroups in the testing (cutoff = 2.012904), entire (cutoff = 1.848551) and the GSE22541 cohorts (cutoff = 21.73013). PCA indicated that patients were well divided into low-risk and high-risk groups (Fig. 5A-C) and the survival rate of the former was significantly higher than that of the latter (Fig. 5D-F). The AUCs of the 1-, 2- and 3-year ROC curves of the testing cohort were 0.735, 0.637 and 0.673, respectively (Fig. 5G). The AUCs of the 1-, 2- and 3-year ROC curves of the entire cohort were 0.729, 0.692 and 0.729, respectively (Fig. 5H). The AUCs of the 1-, 2- and 3-year ROC curves of the GSE22541 cohort were 0.913, 0.730 and 0.722, respectively (Fig. 5I). The number of deaths in the high-risk group was larger than that in the low-risk group, with shorter survival time. There were significant differences in survival time between the high-risk and low-risk groups (Fig. 5J-L).

### Independent prognostic value of the risk model

**Fig. 6:**
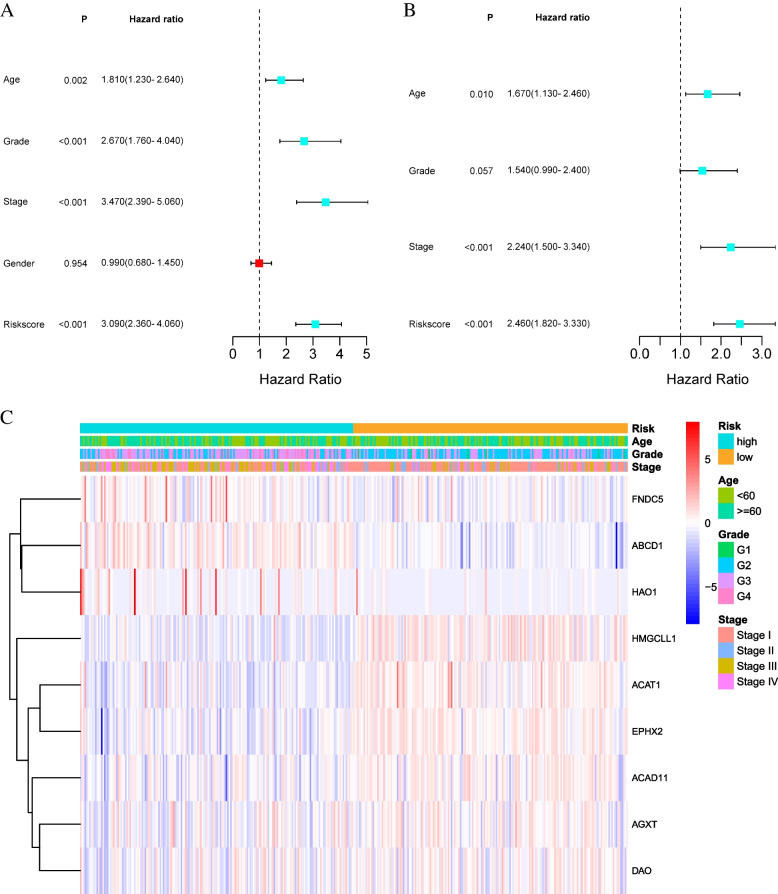
Independent prognostic analysis. **A** Univariate Cox regression analysis in the training cohort. **B** Multivariate Cox regression analysis in the training cohort. **C** Heatmap of the expression of 9 model genes and the distribution of clinical factors between high-risk and low-risk groups

The univariate Cox regression analysis showed the risk score (95%CI: 2.360–4.060; *p* < 0.001), age (95%CI: 1.230–2.640; *p* = 0.002), stage (95%CI: 2.390–5.060; *p* < 0.001) and grade (95%CI: 1.760–4.040; *p* < 0.001) were significantly associated with the OS of KIRC patients, yet gender was not a significant prognostic factor (Fig. 6A). Then multivariate Cox regression analysis showed the risk score (95%CI: 1.820–3.330; *p* < 0.001), age (95%CI: 1.130–2.460; *p* = 0.010) and stage (95%CI: 1.500–3.340; *p* < 0.001) were important and significant prognostic factors (Fig. 6B). The above results indicated that the risk score, age and stage had the ability to be independent risk factors. We plotted a heatmap of the clinical characteristics of the training cohort and found differences in patient age and stage between the low-risk and high-risk groups (Fig. 6C).

### Establishment and validation of the nomogram

**Fig. 7:**
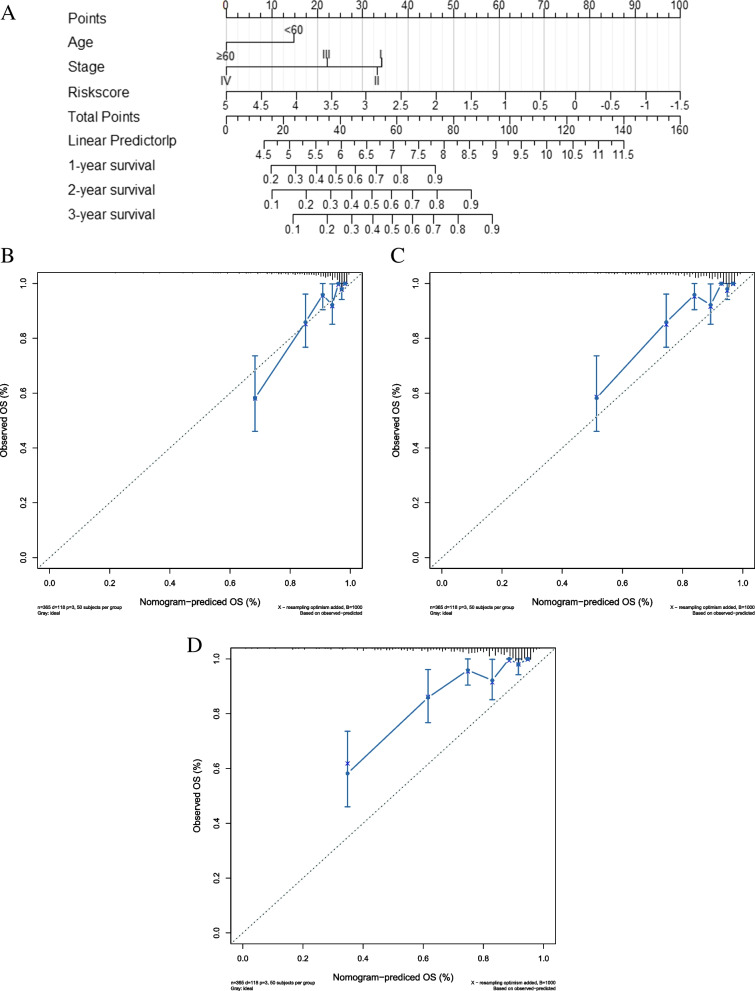
Establishment and Validation of the nomogram. **A** Nomogram to predict the OS of KIRC patients in 1-, 2- and 3- year. **B**-**D** The calibration plots for the training cohort of the nomogram for KIRC patients in 1-, 2- and 3- year. The Y-axis represents actual survival, and the X-axis represents nomogram-predicted survival

The significantly prognostic clinical factors, including age and stage, and risk score were integrated to establish a nomogram, which was used to predict the overall survival rate of 1-year, 2-year and 3-year KIRC patients, using the R package “rms” (Fig. 7A). The C-index of the nomogram was 0.77(0.77 > 0.7). The results of the calibration curve at 1 year were consistent with the actual results, but the calibration curves at 2 years and 3 years deviated from the actual results (Fig. 7B-D).

### GO enrichment and KEGG pathway analyses

**Fig. 8:**
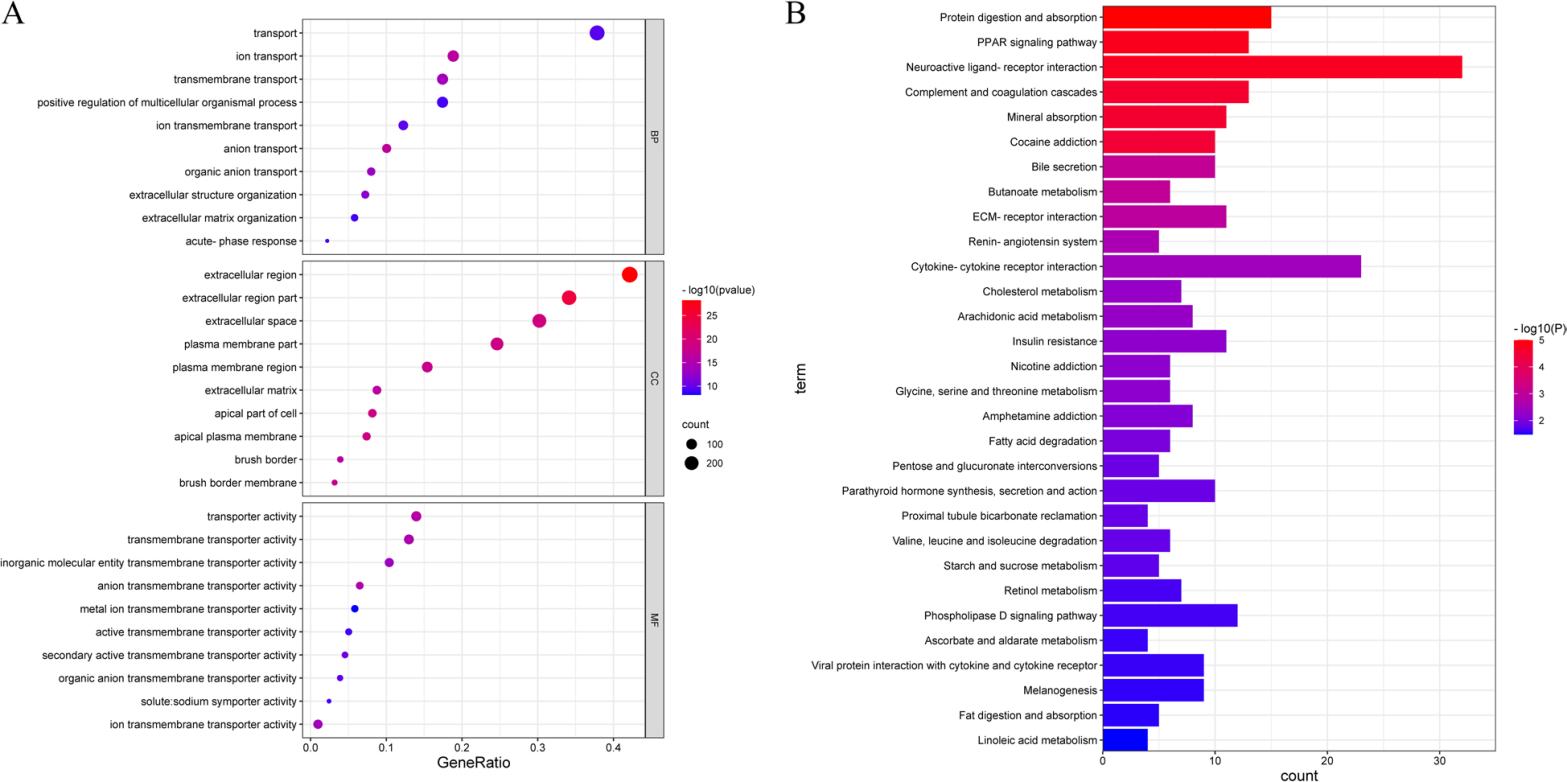
GO enrichment and KEGG pathway analyses. **A** Bubble graph for GO enrichment analysis (the bigger bubble means the more genes enriched, and the increasing depth of red means the differences were more obvious;). **B** Barplot graph for KEGG pathway analysis (the longer bar means the more genes enriched, and the increasing depth of red means the differences were more obvious)

To further explore the differences in gene function and pathways, we used the “limma” package, with *p* < 0.05 and |log_2_ FC| > 1 as criteria for screening DEGs. We screened DEGs in the high-risk group and low-risk group. GO and KEGG analyses results indicated that DEGs were mainly related to substance metabolism, transport and the PPAR signaling pathway (Fig. 8A-B).

### Tumor immune infiltration between two subgroups

**Fig. 9:**
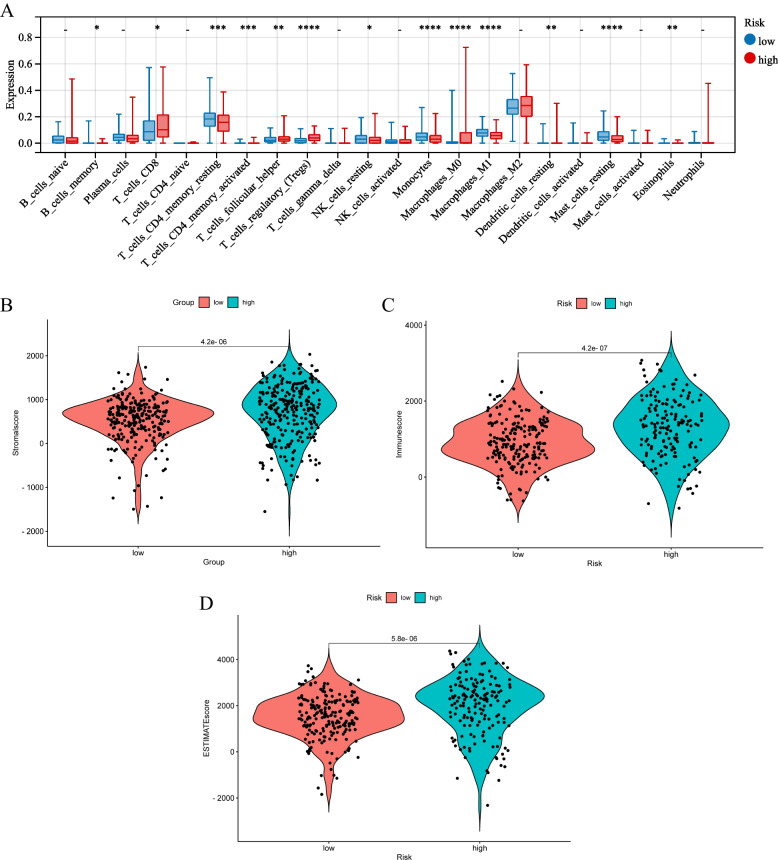
The immune analysis between the high-risk and low-risk groups. **A** Comparison of the scores of 22 types of immune cells between high-risk and low-risk groups in the training cohort. **B**–**D** Expression level of (**B**) Stromal score, (**C**) ESTIMATE score and (**D**) immune score between the high-risk and low-risk groups

**Fig. 10:**
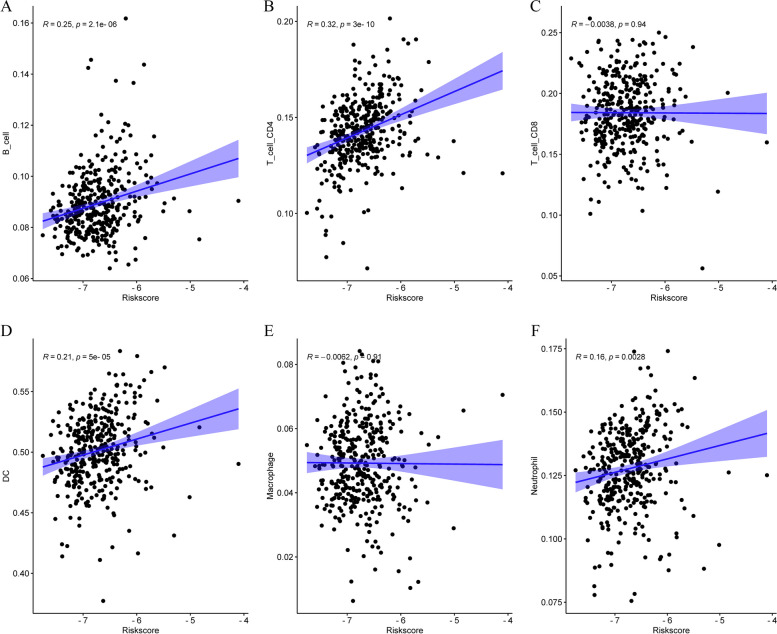
The associations between the risk score and infiltration levels of 6 immune cell types. **A** B cells (**B**) CD4 + T cells (**C**) CD8 + T cells (**D**) DC (**E**)Macrophages and (**F**) Neutrophils.

**Fig. 11:**
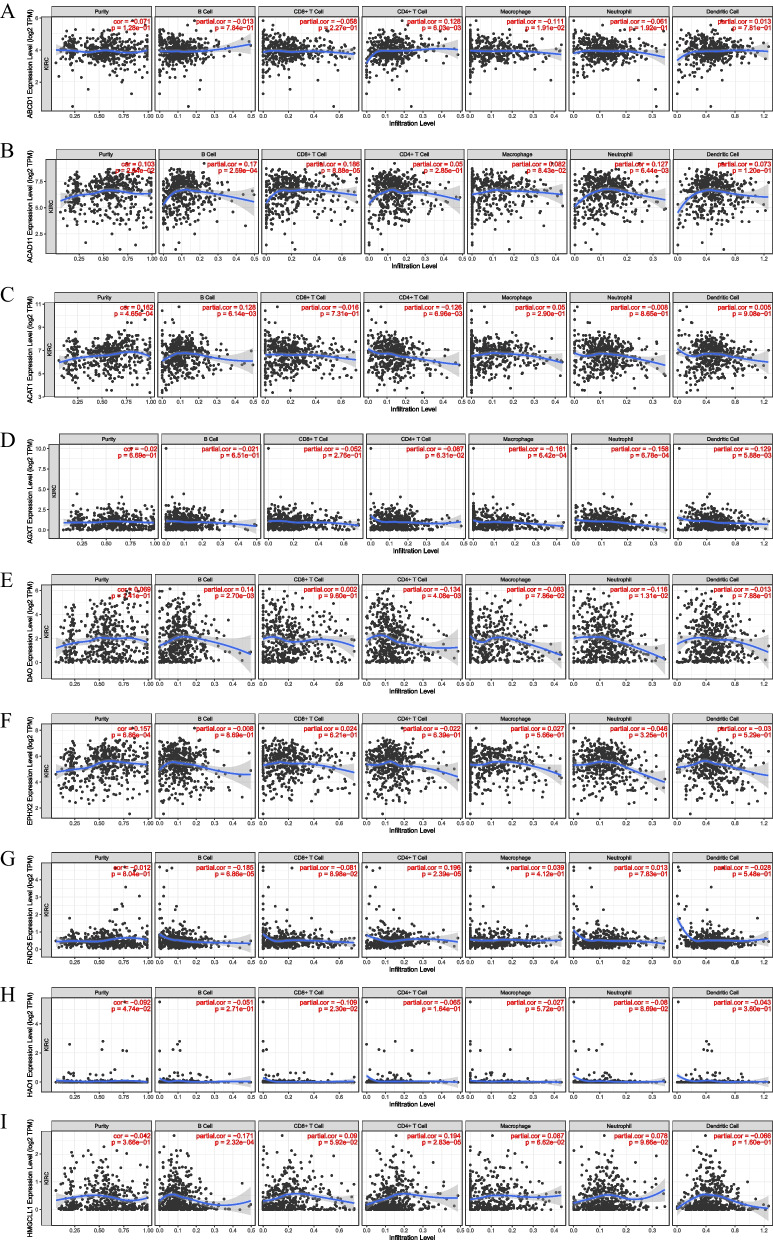
The correlation of 9 model genes expression with immune infiltration levels in KIRC. **A** ABCD1. **B** ACAD11. **C** ACAT1. **D **AGXT. **E** DAO. **F** EPHX2. **G** FNDC5. **H** HAO1. **I** HMGCLL1.

**Fig. 12:**
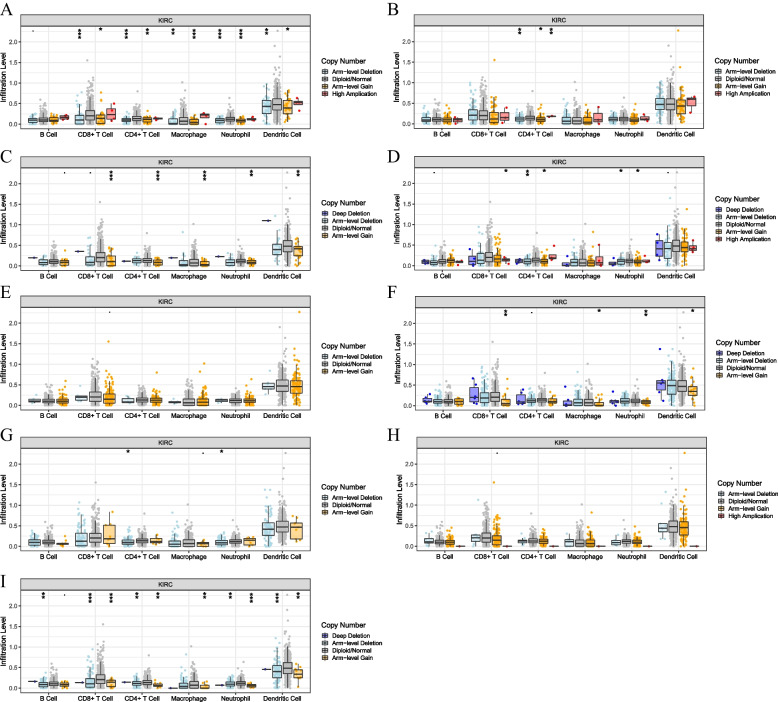
The correlation between somatic copy number alterations of nine genes and the abundance of immune infiltration. **A** ABCD1. **B** ACAD11. **C** ACAT1. **D** AGXT. **E** DAO. **F** EPHX2. **G** FNDC5. **H** HAO1. **I** HMGCLL1

We used CIBERSORT database to compare the infiltration fractions of 22 immune cells in the training cohort. The infiltration levels of CD8^+^ T cells, follicular helper T cells, regulatory T cells, macrophages, stationary dendritic cells and eosinophils were significantly higher in the high-risk group (*p* < 0.05). The infiltration levels of memory B cells, CD4^+^ T cells, monocytes, M1 macrophages and stationary mast cells were significantly higher in the low-risk group (*p* < 0.05). The results indicated that specific and nonspecific immunity were suppressed in the high-risk group (Fig. 9A). To further evaluate the relationship between the immune status of each group, we successfully generated the stromal score, immune score, and ESTIMATE score by using the ESTIMATE algorithm. The special situation is that higher stroma score (*p* < 0.001), immune score (*p* < 0.001), and ESTIMATE score (*p* < 0.001) and higher tumor purity were observed in the high-risk group compared with the low-risk group (Fig. 9B-D). We also analyzed the relationship between the infiltration levels of 6 immune cell types and the risk score by using TIMER database. Except for CD8^+^ T cells and macrophages, there was a significant positive correlation between the risk score and the abundance of other immune cells (Fig. 10A-F). We further analyzed the correlation between the expression of 9 genes and the level of immune infiltration by using TIMER database, and the results indicated that the expression of these genes was positively correlated with the level of immune cell infiltration (Fig. 11A-I). What’s more, we studied the potential correlation between infiltration of 6 immune cell types and the somatic copy number alterations of 9 model genes using the “SCNA” module of TIMER database. Except for DAO and HAO1, the mutants of 7 other genes were strongly related to the immune infiltration microenvironment of KIRC. Specifically, arm-level deletion and arm-level gain had a statistically significant impact on the immune cell infiltration levels of KIRC (Fig. 12A-I).

### Identification of potential prognostic biomarkers

Using GEPIA2.0 website and based on TCGA database, we conducted survival analysis on 9 model genes. Employing the median expression as the cutoff value, we categorized KIRC patients into high and low expression groups. Through meticulous screening, a compelling observation surfaced: in KIRC patients, those with high expression levels of ACAT1, EPHX2, and HMGCL11 exhibited superior OS and RFS compared to their low-expression group (Fig. S2A-F). Upon this discovery, we proceeded to construct a nomogram representing the expression patterns of these three genes. Notably, the segment corresponding to ACAT1 stood out with the longest length, indicating its superior prognostic capabilities in predicting the outcomes of KIRC patients compared to EPHX2 and HMGCL11 (Fig. S2G). This underscores the potential clinical significance of ACAT1 as a potential prognostic marker in the context of KIRC. Subsequent analysis of ACTA1 protein expression using the UALCAN database revealed that ACAT1 protein levels were lower in KIRC than in normal tissues (Fig. S2H). Further validation through the HPA database, focusing on both normal and KIRC tissue microarrays, affirmed this trend, illustrating an overall lower expression level of ACAT1 protein in KIRC tumor tissues compared to their normal tissues (Fig. S2I).

### Immunohistochemistry

**Fig. 13:**
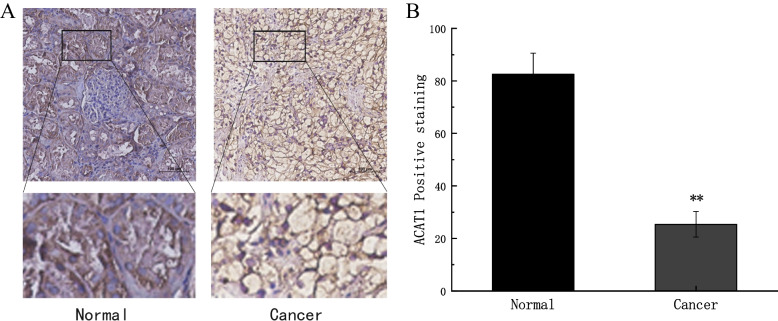
Protein expression level of ACAT1 KIRC and normal tissues were examined via immunohistochemical staining

The potential clinical significance of the ACAT1 protein was preliminarily assessed by IHC in KIRC patients from Huaihe Hospital of Henan University. The IHC analysis showed that ACAT1 expression was predominantly located in the cell membrane and cytoplasm of renal tubular epithelial cells, and notably we found that ACAT1 protein expression was significantly down-expressed in KIRC tissues compared with normal tissues (*p <* 0.01, Fig. 13A-B).

## Discussion

KIRC is a malignant tumor originating from the epithelial cells of renal tubules, and its progression is closely linked to a profound restructuring of cellular metabolism, and the metabolic features of KIRC are primarily manifested through the reprogramming of energy metabolism [13–15]. In order to meet the demands of their rapid growth and proliferation, cells undergo a series of intricate metabolic regulations. Specifically, glycolysis, as a crucial pathway for ATP generation in cells, is widely activated in KIRC, which provides the required energy for cells by breaking down glucose into pyruvate and lactate. However, compared to normal cells, KIRC cells exhibit a significantly higher degree of dependence on this pathway, resulting in a noticeable increase in glycolytic flux [16–18]. Simultaneously, mitochondria, as the primary organelles for intracellular energy production, also play a critical role in the metabolic processes of renal cell carcinoma. Research indicates that mitochondrial bioenergetics in renal cell carcinoma undergo significant alterations, leading to impaired oxidative phosphorylation (OxPhox) function. This phenomenon may be a result of cells adjusting mitochondrial function to meet the demands of rapid proliferation, prioritizing ATP production through glycolysis rather than relying on oxidative phosphorylation [19]. In the metabolic reprogramming of KIRC, lipid metabolism also plays a crucial role. The demand for lipids becomes increasingly significant in cancer cell growth and division, and KIRC cells exhibit abnormal reliance on lipids. Significant changes occur in the synthesis and breakdown of lipids during this process, influencing the structure and function of cell membranes [17, 20]. Peroxisomes, as organelles involved in lipid metabolism and cellular redox balance, play a crucial role in the metabolic regulation of KIRC.

The immune infiltrative nature of KIRC has drawn widespread attention, highlighting the significant role of the immune system in tumor development. KIRC stands out due to its immune infiltrative characteristics, typically involving various immune cells such as T lymphocytes, natural killer cells, and macrophages, all playing anti-tumor roles in the tumor microenvironment. Understanding the molecular mechanisms of immune infiltration in KIRC is crucial for formulating effective immunotherapeutic strategies [21, 22]. On the metabolic front, the activation of specific metabolic pathways plays a pivotal role in the immune infiltration of KIRC. Some studies suggest a close relationship between the activation of the glycolytic pathway and the infiltration and functionality of immune cells. Tumor cells influence the metabolic environment of immune cells by regulating the generation of glycolytic products, thereby modulating the activity and function of immune cells. Additionally, certain metabolic byproducts may directly impact the acid-base balance and oxygen concentration in the tumor microenvironment, thereby influencing immune cell infiltration. Vascularization also plays a crucial role in the development of KIRC. The generation of intratumoral blood vessels is closely related to tumor growth and metastasis, with the activation of specific metabolic pathways regulating angiogenesis and inflammatory features [23, 24]. Furthermore, the characteristics of the tumor microenvironment significantly impact the disease biology of KIRC. The tumor microenvironment comprises various components such as cytokines, growth factors, extracellular matrix, etc., and their interactions with immune cells have a crucial impact on the effectiveness of immunotherapy [24–27]. In-depth exploration of the composition and regulatory mechanisms of the KIRC microenvironment is instrumental in gaining a better understanding of the molecular basis of tumor development, thereby providing more precise targets for individualized treatment. Peroxisomes, as metabolic organelles within cells, also play a crucial role in the immune regulation of KIRC. By modulating lipid metabolism and redox balance, peroxisomes influence the activity and infiltration of immune cells in renal cell carcinoma. Further investigation into the functions of peroxisomes in KIRC holds the potential to offer new insights for the development of novel immune therapy strategies.

In this study, the Cox regression analyses were applied to identify 14 significantly prognostic DE-PRGs, subsequently, utilizing the advanced LASSO Cox regression analysis, we refined these genes, ultimately narrowing it down to a subset of 9 critical genes. The predictive capacity of gene risk model was evaluated in thorough validation cohorts, including training, testing, entire and GSE22541 cohorts, and the results showed both its better accuracy and sensitivity. Notably, independent prognosis analyses unequivocally demonstrated that the risk scores derived from gene risk model possess the potential to act as independent prognostic factors for patients with KIRC. We also employed a holistic approach by integrating survival time, survival status, age, stage, and risk score to construct a nomogram. The C-index was 0.77 which showed good predictive performance. While the 1-year calibration curve aligned closely with actual results, observed discrepancies in the 2-year and 3-year calibration curves prompted a critical examination. These deviations are tentatively attributed to the inherent limitations posed by a relatively small sample size, necessitating a cautious interpretation of these specific time points. Furthermore, our investigation delved into the realm of immune responses, revealing intriguing insights. Multiple immune analyses unearthed higher tumor purity within the high-risk group, concurrently accompanied by suppressed immune reactions. This nuanced observation offers a plausible explanation for the observed poorer prognosis within the high-risk group, as elucidated in the survival curve. The intricate interplay between genetic factors, immune responses, and clinical parameters emerges as a focal point, underscoring the complexity of prognostic modeling in KIRC. Through the analyses of 9 model genes, including univariate and multivariate Cox regression, survival and nomogram analysis, our attention was eventually honed in on ACAT1. These integrated findings not only highlight the prognostic significance of ACAT1 but also underscore the consistency of its downregulation at both the mRNA and protein levels in KIRC. The multiple analyses, incorporating survival analysis and protein expression validation, enhance the robustness of our observations, providing a comprehensive understanding of ACAT1’s potential role in the clinical landscape of KIRC. Further exploration into the molecular mechanisms governing ACAT1 expression may unveil novel therapeutic avenues for managing KIRC patients. This study was based on data mining from the TCGA and GEO databases. The initial validation was conducted using clinical specimens for immunohistochemistry. However, extensive follow-up experiments are essential, including PCR, Western Blot, CCK-8 and Transwell, etc., to validate the role of ACAT1 in the mechanism of action in KIRC. These experiments are crucial for providing new therapeutic strategies towards personalized treatment approaches.

ABCD1 holds significance in X-linked adrenal leukodystrophy (X-ALD) as it plays a pivotal role in facilitating the entry of very-long-chain fatty acids (VLCFAs) into the peroxisome for subsequent β-oxidation [28]. Situated within the peroxisomal membrane, ABCD1 orchestrates the breakdown of VLCFAs via β-oxidation by transporting cytoplasmic VLCFAs or VLCFA-CoA into the peroxisome [29]. Another vital gene, ACAD11, encodes a protein deeply involved in fatty acid oxidation, a process critical for efficient oxidative phosphorylation (OXPHOS) and cellular survival, especially under glucose deprivation conditions [30]. Considering the frequently observed metabolic shift in cancer development characterized by escalated aerobic glycolysis and diminished OXPHOS [30]. ACAD11 assumes importance. Notably, it emerges as a pivotal metabolic target of the tumor suppressor protein p53, known for its role in inhibiting tumor progression by curtailing glycolytic activity and fostering OXPHOS through diverse mechanisms [31]. Therefore, ACAD11 potentially plays a crucial role in the context of tumor advancement. ACAT contains two forms: one is a cytoplasmic enzyme (ACAT2), and the other is a mitochondrial enzyme (ACAT1), which could catalyze the reversible formation of acetyl-CoA from two molecules in the process of ketogenesis and ketolysis, respectively. Recent studies have revealed a potential carcinogenic impact associated with ACAT1, in which overexpression of ACAT1 contributes to promoting tumor growth and metastasis, lending support to the hypothesis implicating key enzymes involved in ketone body metabolism in the process of tumorigenesis and metastatic progression [32, 33]. AGXT, also recognized as AGT, encodes a liver peroxisomal enzyme responsible for catalyzing the conversion of glyoxylic acid to glycine. The inactivation of AGXT protein results in the conversion of glyoxylic acid to oxalate, leading to the formation of insoluble calcium salt deposits primarily in the kidney and other organs [34]. Primary hyperoxaluria type 1 (PH1) is an uncommon metabolic disorder characterized by defects in liver-specific peroxisome enzymes, specifically alanine-acetaldehyde acid and serine-pyruvate aminotransferase [35]. The DAO gene, responsible for encoding diamine oxidase, has been linked to allergic reactions, and intriguingly, mutations in this gene might contribute to the incidence of gastric cancer [36]. EPHX2, encoding soluble epoxide hydrolase (seH), plays a crucial role in the degradation of endogenous lipid epoxides [37]. Dysregulation of EPHX2 has been implicated in various diseases, including renal and liver malignancies [38], hypertension [39], and hypercholesterolemia [40]. FNDC5, a transmembrane glycoprotein released during muscle cell activity, produces irisin upon hydrolysis. Irisin, in turn, responds to the activation of peroxisome proliferator-activated receptor γ coactivator 1α (PGC-1α) [41, 42]. This multifunctional hormone has implications in metabolism, diabetes, cardiovascular diseases [43], and has also been associated with the occurrence and development of cancer [44]. HAO1, predominantly expressed in the liver and pancreas, exhibits activity against the dicarbon substrate glycolate [45]. Recent studies highlight its role in regulating tricarboxylic acid (TCA) circulation [46]. Targeted therapy of the HAO1 gene holds promise for addressing hyperoxaluria. HMGCLL1, a highly homologous gene to HMGCL reported in the Genome Database in 2004, encodes the lytic isoform HL (ER-CHL), capable of generating acetoacetic acid and acetyl-CoA. Notably, studies indicate a decline in HMGCLL1 expression levels in KIRC [47]. Understanding the intricate relationships between these genes and their associated pathways is crucial for unraveling the complexities of metabolic disorders and developing effective therapeutic strategies. This emerging body of evidence emphasizes the multifaceted involvement of peroxisomes in cancer, specifically KIRC. Understanding the nuanced interplay between peroxisomes and tumorigenesis offers potential avenues for targeted interventions and therapeutic strategies. The intricate relationship between peroxisomal function and the molecular intricacies of KIRC underscores the need for further exploration in this intriguing field of research.

In conclusion, our research not only sheds light on the potential prognostic significance of peroxisome-related genes in KIRC but also contributes valuable insights into the molecular and immune landscape of this challenging malignancy. The establishment of a nine-gene prognostic risk model, combined with functional and immune analyses, forms the basis for a comprehensive approach towards personalized treatment strategies for KIRC. This study aims to bridge existing gaps in prognostic evaluation and therapeutic decision-making, ultimately improving outcomes for patients with KIRC.

### Supplementary Information

**Additional file 1: Table S1.** Characteristics of KIRC patients in this study. **Table S2.** 113 peroxisome -related genes. **Fig. S1.** Multivariate Cox regression analysis. **Fig. S2.** Identification of potential prognostic biomarkers. (A-F) Kaplan-Meier curves of ACAT, EPHX2, and HMGCLL1 in KIRC. (G) Nomogram of ACAT1, EPHX2 and HMGCLL1. (H) Protein expression of ACAT1 in KIRC and normal groups in UALCAN database. (I) HPA database.

## Data Availability

The datasets generated and/or analyzed during the current study are available in the UCSC xena (http://xena.ucsc.edu/) and GEO (https://www.ncbi.nlm.nih.gov/).

## Abbreviations

KIRC: Kidney clear cell carcinoma
pRCC: Papillary renal cell carcinoma
chRCC: Chromophobe cell carcinoma
DE-PRGs: Differential expressed peroxisome-related genes
UCSC: University of Cingifornia Sisha Cruz
TCGA: The Cancer Genome Atlas
GEO: Gene Expression Omnibus
LASSO: Least absolute shrinkage and selection operator
GO: Gene ontology
KEGG: Kyoto Encyclopedia of Genes and Genomes
CIBERSORT: Cell-type Identification By Estimating Relative Subsets Of RNA Transcripts
ESTIMATE: Estimation of Stromal and Immune cells in Malignant Tumor tissues using Expression data
TIMER: Tumor Immune Estimation Resource
PCA: Principal component analysis
ROC: Receiver operating characteristic
AUC: Area under the curve
OS: Overall Survival
SCNA: Somatic copy number variation
