# Supplementary material for: Establishment and validation of a novel peroxisome-related gene prognostic risk model in kidney clear cell carcinoma
Source: BMC Urol. 2024 Jan 31;24:26. doi: 10.1186/s12894-024-01404-z (PMC10829319; doi:10.1186/s12894-024-01404-z)
Supplement: Supplementary file 1 — Additional file 1: Table S1. Characteristics of KIRC patients in this study. Table S2. 113 peroxisome -related genes. Fig. S1. Multivariate Cox regression analysis. Fig. S2. Identification of potential prognostic biomarkers. (A-F) Kaplan-Meier curves of ACAT, EPHX2, and HMGCLL1 in KIRC. (G) Nomogram of ACAT1, EPHX2 and HMGCLL1. (H) Protein expression of ACAT1 in KIRC and normal groups in UALCAN database. (I) HPA database. [file 12894_2024_1404_MOESM1_ESM.docx]

| **Table S1 Characteristics of KIRC patients in this study** | | | | |
| --- | --- | --- | --- | --- |
| Characteristics | Training(n=365) | Testing(n=157) | Entire(n=522) | GSE22541(n=24) |
| **Age** |  |  |  |  |
| <60 | 167(31.99%) | 76(14.56%) | 243(46.55%) |  |
| ≥60 | 198(37.93%) | 81(15.52%) | 279(53.45%) |  |
| **Gender** |  |  |  |  |
| Female | 119(22.80%) | 63(12.07%) | 182(34.87%) | 11(45.83%) |
| Male | 246(47.13%) | 94(18.01%) | 340(65.13%) | 13(54.17%) |
| **Grade** |  |  |  |  |
| G1 | 8(1.53%) | 5(0.96%) | 13(2.49%) |  |
| G2 | 165(31.61%) | 63(12.07%) | 228(43.68%) | 18(75.00%) |
| G3 | 141(27.01%) | 65(12.45%) | 206(39.46%) | 6(25.00%) |
| G4 | 51(9.77%) | 24(4.60%) | 75(14.37%) |  |
| **Stage** |  |  |  |  |
| Stage I | 183(35.06%) | 79(15.13%) | 262(50.19%) |  |
| Stage II | 40(7.66%) | 14(2.68%) | 54(10.34%) |  |
| Stage III | 88(16.86%) | 34(6.51%) | 122(23.37%) |  |
| Stage IV | 54(10.34%) | 30(5.75%) | 84(16.09%) |  |
| **Status** |  |  |  |  |
| Dead | 117(22.41%) | 56(10.73%) | 173(33.14%) | 6(25.00) |
| Alive | 248(47.51%) | 101(19.35%) | 349(66.86%) | 18(75.00%) |

**Table S2** 113 peroxisome -related genes

| **Gene symbol** | **Gene Description** |
| --- | --- |
| ABCD1 | ATP binding cassette subfamily D member 1 |
| ABCD2 | ATP binding cassette subfamily D member 2 |
| ABCD3 | ATP binding cassette subfamily D member 3 |
| ABCD4 | ATP binding cassette subfamily D member 4 |
| ACAA1 | acetyl-CoA acyltransferase 1 |
| ACAD11 | acyl-CoA dehydrogenase family member 11 |
| ACAT1 | acetyl-CoA acetyltransferase 1 |
| ACBD5 | acyl-CoA binding domain containing 5 |
| ACOT1 | acyl-CoA thioesterase 1 |
| ACOT2 | acyl-CoA thioesterase 2 |
| ACOT4 | acyl-CoA thioesterase 4 |
| ACOT8 | acyl-CoA thioesterase 8 |
| ACOX1 | acyl-CoA oxidase 1 |
| ACOX2 | acyl-CoA oxidase 2 |
| ACOX3 | acyl-CoA oxidase 3, pristanoyl |
| ACSF3 | acyl-CoA synthetase family member 3 |
| ACSL1 | acyl-CoA synthetase long chain family member 1 |
| ACSL3 | acyl-CoA synthetase long chain family member 3 |
| ACSL4 | acyl-CoA synthetase long chain family member 4 |
| ACSL5 | acyl-CoA synthetase long chain family member 5 |
| ACSL6 | acyl-CoA synthetase long chain family member 6 |
| ADH1A | alcohol dehydrogenase 1A (class I), alpha polypeptide |
| AGPS | alkylglycerone phosphate synthase |
| AGXT | alanine--glyoxylate and serine--pyruvate aminotransferase |
| ALDH3A2 | aldehyde dehydrogenase 3 family member A2 |
| AMACR | alpha-methylacyl-CoA racemase |
| ATAD1 | ATPase family AAA domain containing 1 |
| BAAT | bile acid-CoA: amino acid N-acyltransferase |
| CAT | catalase |
| CRAT | carnitine O-acetyltransferase |
| CROT | carnitine O-octanoyltransferase |
| DAO | D-amino acid oxidase |
| DDO | D-aspartate oxidase |
| DECR2 | 2,4-dienoyl-CoA reductase 2 |
| DHRS4 | dehydrogenase/reductase 4 |
| DHRS4L1 | dehydrogenase/reductase 4 like 1 |
| DNAJC10 | DnaJ heat shock protein family (Hsp40) member C10 |
| DNM1L | dynamin 1 like |
| ECH1 | enoyl-CoA hydratase 1 |
| ECI2 | enoyl-CoA delta isomerase 2 |
| EHHADH | enoyl-CoA hydratase and 3-hydroxyacyl CoA dehydrogenase |
| EPHX2 | epoxide hydrolase 2 |
| FAR1 | fatty acyl-CoA reductase 1 |
| FAR2 | fatty acyl-CoA reductase 2 |
| FIS1 | fission, mitochondrial 1 |
| FNDC5 | fibronectin type III domain containing 5 |
| GNPAT | glyceronephosphate O-acyltransferase |
| GRHPR | glyoxylate and hydroxypyruvate reductase |
| GSTK1 | glutathione S-transferase kappa 1 |
| HACL1 | 2-hydroxyacyl-CoA lyase 1 |
| HAO1 | hydroxyacid oxidase 1 |
| HAO2 | hydroxyacid oxidase 2 |
| HMGCL | 3-hydroxy-3-methylglutaryl-CoA lyase |
| HMGCLL1 | 3-hydroxymethyl-3-methylglutaryl-CoA lyase like 1 |
| HSD17B4 | hydroxysteroid 17-beta dehydrogenase 4 |
| IDE | insulin degrading enzyme |
| IDH1 | isocitrate dehydrogenase (NADP (+)) 1 |
| IDH2 | isocitrate dehydrogenase (NADP (+)) 2 |
| IDI1 | isopentenyl-diphosphate delta isomerase 1 |
| IDI2 | isopentenyl-diphosphate delta isomerase 2 |
| ISOC1 | isochorismatase domain containing 1 |
| LDHA | lactate dehydrogenase A |
| LONP2 | Lon peptidase 2, peroxisomal |
| MDH1 | malate dehydrogenase 1 |
| MLYCD | malonyl-CoA decarboxylase |
| MPV17 | mitochondrial inner membrane protein MPV17 |
| MPV17L | MPV17 mitochondrial inner membrane protein like |
| MPV17L2 | MPV17 mitochondrial inner membrane protein like 2 |
| MTARC2 | mitochondrial amidoxime reducing component 2 |
| MVK | mevalonate kinase |
| NOS2 | nitric oxide synthase 2 |
| NUDT12 | nudix hydrolase 12 |
| NUDT19 | nudix hydrolase 19 |
| NUDT7 | nudix hydrolase 7 |
| PAOX | polyamine oxidase |
| PECR | peroxisomal trans-2-enoyl-CoA reductase |
| PEX1 | peroxisomal biogenesis factor 1 |
| PEX10 | peroxisomal biogenesis factor 10 |
| PEX11A | peroxisomal biogenesis factor 11alpha |
| PEX11B | peroxisomal biogenesis factor 11beta |
| PEX11G | peroxisomal biogenesis factor 11gamma |
| PEX12 | peroxisomal biogenesis factor 12 |
| PEX13 | peroxisomal biogenesis factor 13 |
| PEX14 | peroxisomal biogenesis factor 14 |
| PEX16 | peroxisomal biogenesis factor 16 |
| PEX19 | peroxisomal biogenesis factor 19 |
| PEX2 | peroxisomal biogenesis factor 2 |
| PEX26 | peroxisomal biogenesis factor 26 |
| PEX3 | peroxisomal biogenesis factor 3 |
| PEX5 | peroxisomal biogenesis factor 5 |
| PEX5L | peroxisomal biogenesis factor 5 like |
| PEX6 | peroxisomal biogenesis factor 6 |
| PEX7 | peroxisomal biogenesis factor 7 |
| PHYH | phytanoyl-CoA 2-hydroxylase |
| PIPOX | pipecolic acid and sarcosine oxidase |
| PMVK | phosphomevalonate kinase |
| POMC | proopiomelanocortin |
| PRDX1 | peroxiredoxin 1 |
| PRDX5 | peroxiredoxin 5 |
| PXMP2 | peroxisomal membrane protein 2 |
| PXMP4 | peroxisomal membrane protein 4 |
| RHOC | ras homolog family member C |
| SCP2 | sterol carrier protein 2 |
| SERHL | serine hydrolase like (pseudogene) |
| SLC22A5 | solute carrier family 22 member5 |
| SLC25A17 | solute carrier family 25 member17 |
| SLC27A2 | solute carrier family 27 member2 |
| SOD1 | superoxide dismutase 1 |
| SOD2 | superoxide dismutase 2 |
| TMEM135 | transmembrane protein 135 |
| TRIM37 | tripartite motif containing 37 |
| XDH | xanthine dehydrogenase |
| ZADH2 | zinc binding alcohol dehydrogenase domain containing 2 |


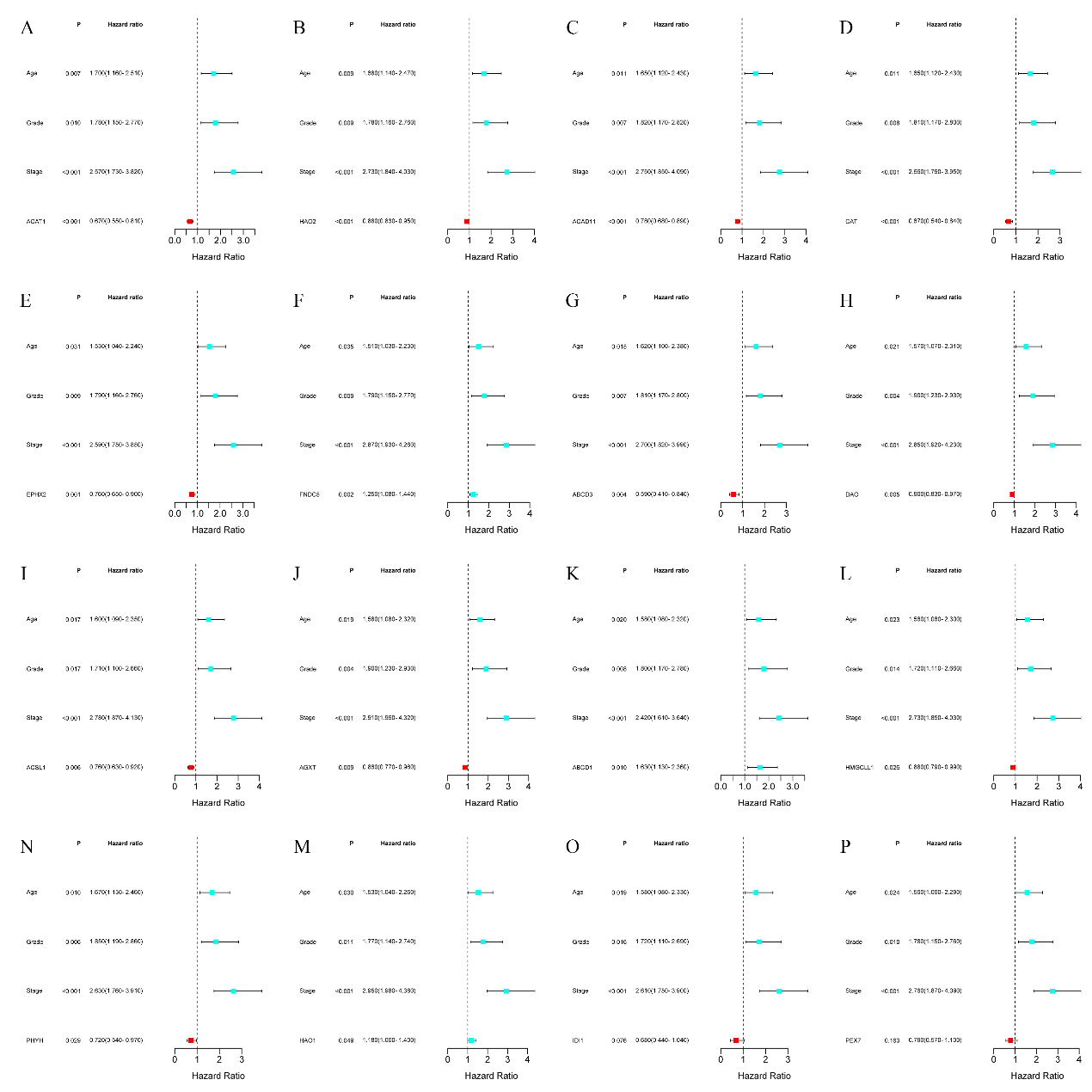


**Fig. S1**. Multivariate Cox regression analysis.


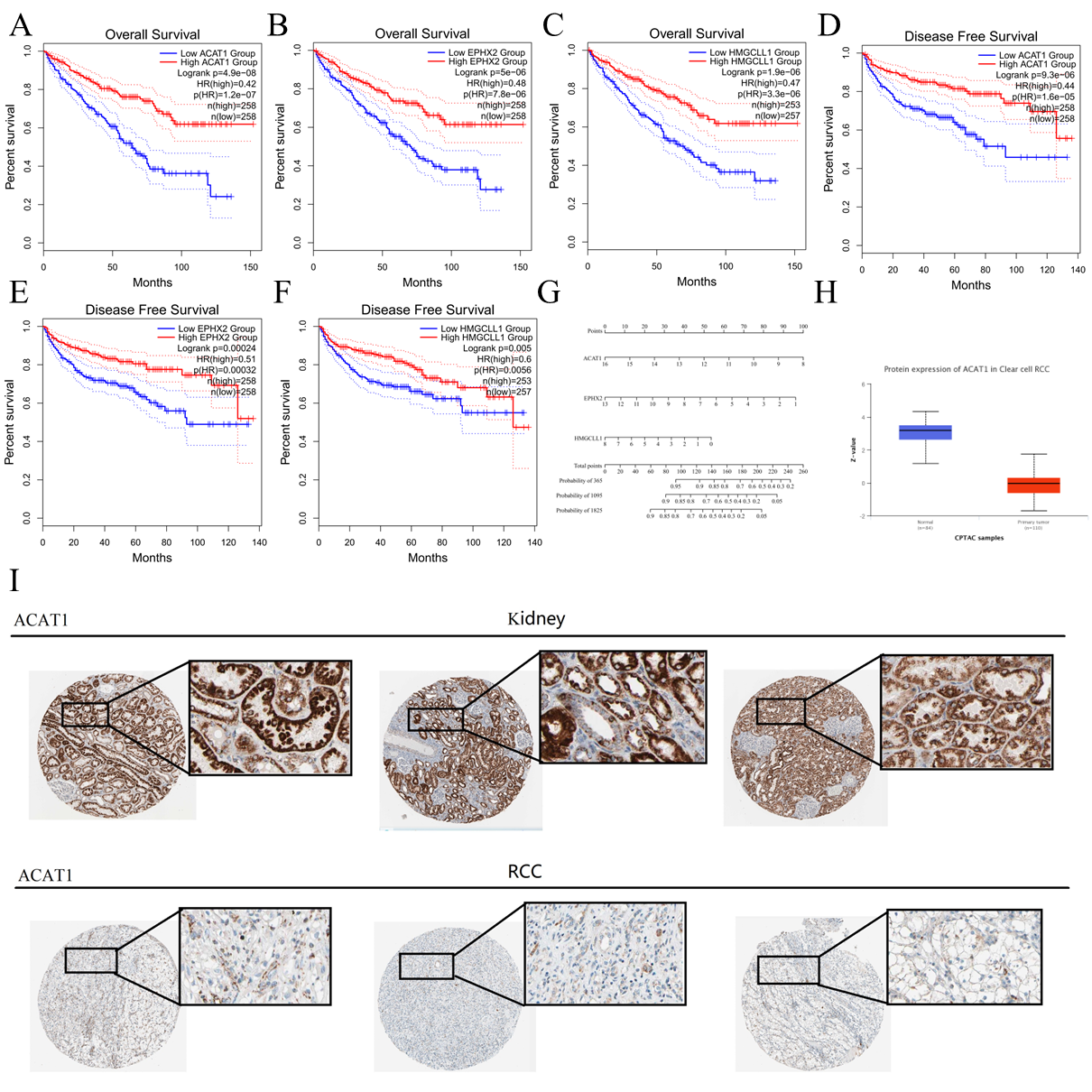


**Fig. S2** Identification of potential prognostic biomarkers. (A-F) Kaplan-Meier curves of ACAT, EPHX2, and HMGCLL1 in KIRC. (G) Nomogram of ACAT1, EPHX2 and HMGCLL1. (H) Protein expression of ACAT1 in KIRC and normal groups in UALCAN database. (I) HPA database.
